# Tight perioperative glucose control is associated with a reduction in renal impairment and renal failure in non-diabetic cardiac surgical patients

**DOI:** 10.1186/cc7145

**Published:** 2008-12-04

**Authors:** Patrick Lecomte, Bruno Van Vlem, Jose Coddens, Guy Cammu, Guy Nollet, Frank Nobels, Hugo Vanermen, Luc Foubert

**Affiliations:** 1 Department of Anaesthesiology and Critical Care Medicine, Onze-Lieve-Vrouw Hospital, Moorselbaan 164, 9300 Aalst, Belgium; 2 Department of Nephrology, Onze-Lieve-Vrouw Hospital, Moorselbaan 164, 9300 Aalst, Belgium; 3 Department of Endocrinology, Onze-Lieve-Vrouw Hospital, Moorselbaan 164, 9300 Aalst, Belgium; 4 Department of Cardiothoracic and Vascular Surgery, Onze-Lieve-Vrouw Hospital, Moorselbaan 164, 9300 Aalst, Belgium

## Abstract

**Introduction:**

Acute renal failure after cardiac surgery increases in-hospital mortality. We evaluated the effect of intra- and postoperative tight control of blood glucose levels on renal function after cardiac surgery based on the Risk, Injury, Failure, Loss, and End-stage kidney failure (RIFLE) criteria, and on the need for acute postoperative dialysis.

**Methods:**

We retrospectively analyzed two groups of consecutive patients undergoing cardiac surgery with cardiopulmonary bypass between August 2004 and June 2006. In the first group, no tight glycemic control was implemented (Control, n = 305). Insulin therapy was initiated at blood glucose levels > 150 mg/dL. In the group with tight glycemic control (Insulin, n = 745), intra- and postoperative blood glucose levels were targeted between 80 to 110 mg/dL, using the Aalst Glycemia Insulin Protocol. Postoperative renal impairment or failure was evaluated with the RIFLE score, based on serum creatinine, glomerular filtration rate and/or urinary output. We used the Cleveland Clinic Severity Score to compare the predicted vs observed incidence of acute postoperative dialysis between groups.

**Results:**

Mean blood glucose levels in the Insulin group were lower compared to the Control group from rewarming on cardiopulmonary bypass onwards until ICU discharge (p < 0.0001). Median ICU stay was 2 days in both groups. In non-diabetics, strict perioperative blood glucose control was associated with a reduced incidence of renal impairment (p = 0.01) and failure (p = 0.02) scoring according to RIFLE criteria, as well as a reduced incidence of acute postoperative dialysis (from 3.9% in Control to 0.7% in Insulin; p < 0.01). The 30-day mortality was lower in the Insulin than in the Control group (1.2% vs 3.6%; p = 0.02), representing a 70% decrease in non-diabetics (p < 0.05) and 56.1% in diabetics (not significant). The observed overall incidence of acute postoperative dialysis was adequately predicted by the Cleveland Clinic Severity Score in the Control group (p = 0.6), but was lower than predicted in the Insulin group (1.2% vs 3%, p = 0.03).

**Conclusions:**

In non-diabetic patients, tight perioperative blood glucose control is associated with a significant reduction in postoperative renal impairment and failure after cardiac surgery according to the RIFLE criteria. In non-diabetics, tight blood glucose control was associated with a decreased need for postoperative dialysis, as well as 30-day mortality, despite of a relatively short ICU stay.

## Introduction

Postoperative deterioration of renal function after cardiac surgery remains a serious complication, associated with increased length of Intensive Care Unit (ICU) stay, increased in-hospital morbidity and mortality and with worse long-term outcome [1,2]. Acute renal failure develops in 5% to 30% of cardiac surgical patients depending on its definition, whereas 1% to 5% of them need hemodialysis [1-3]. The need for postoperative renal replacement therapy is an independent risk factor of death [1]. To date, no drug has been identified as truly nephroprotective in cardiac surgical patients. However, tight glycemic control in the ICU is reported to improve morbidity, mortality and outcome in cardiac surgical patients and to reduce the need for postoperative renal replacement therapy by up to 40% [4-6]. Recently, several studies focused on the benefit of intraoperative tight glycemic control and its relationship with postoperative acute renal failure requiring dialysis [3,5-7]. In cardiac surgery poor intraoperative glycemic control in diabetics is associated with a sevenfold increase in postoperative renal failure, whereas severe hyperglycemia during cardiopulmonary bypass (CPB) in non-diabetics is associated with acute renal failure requiring dialysis [3-6]. Recent observations indicate that hyperglycemia-induced oxidative stress inhibits Na^+^/glucose cotransporter activity in renal proximal tubule cells and stimulates renal oxygen consumption by increased endothelial nitric oxide synthase [8,9].

Until recently, the outcome parameter of choice when evaluating the effect of tight glycemic control in cardiac surgical patients has been the incidence of postoperative dialysis. The possible benefit of intra- and postoperative tight glycemic control on the development of renal impairment with elevated creatinine levels and/or decreased glomerular filtration rates, but without the need for renal replacement therapy, is unknown.

Therefore, we evaluated the effect of both intra- and postoperative tight blood glucose control (80 to 110 mg/dL) with continuous intravenous insulin on the incidence and severity of acute kidney injury after cardiac surgery, using the RIFLE criteria. RIFLE is the acronym for R(isk of renal failure), I(njury to kidney function) and F(ailure of kidney function), L(oss of kidney function) and E(nd-stage renal failure) (the criteria are shown in detail in Table 1). According to the consensus criteria of the Acute Dialysis Quality Initiative Workgroup [10], postoperative renal impairment or renal failure was based on the RIFLE criteria and on the need for acute postoperative dialysis. The RIFLE score was recently validated in cardiac surgical patients [11]. We also used the Cleveland Clinic Severity Score to compare the predicted vs observed incidence of postoperative acute renal failure requiring dialysis in both groups [12].

**Table 1:** Overview of the RIFLE criteria [9]

	GFR criteria	Urinary output (UO) criteria
R(isk)	Increased serum creatinine × 1.5 or GFR decrease > 25%	UO < 0.5 mL/kg/u × 6 h
I(njury)	increased serum creatinine × 2 or GFR decrease > 50%	UO < 0.5 mL/kg/u × 12 h
F(ailure)	increased serum creatinine × 3, GFR decrease 75% or serum creatinine ≥ 4 mg/dL	UO < 0.3 mL/kg/u × 24 h or anuria × 12 h
L(oss)	Persistent ARF = complete loss of kidney function > 4 weeks	
E(nd-stage kidney failure)	End stage kidney disease	

ARF, acute renal failure; GFR, glomerular filtration rate.

## Materials and methods

Between August 2004 and June 2006, a total of 1,862 patients were scheduled for cardiac procedures at the Onze-Lieve-Vrouw Hospital in Aalst, Belgium. Inclusion criteria were an age > 18 years and the use of CPB. Exclusion criteria were any surgery needing deep hypothermic circulatory arrest, as well as preoperative end-stage renal failure requiring hemodialysis. All data were retrieved from patient files and from the database of the Department of Cardiothoracic and Vascular Surgery. This study was approved by the hospital ethics committee, and informed consent was waived. Patients not previously treated for diabetes mellitus but with a fasting glucose < 125 mg/dL were considered to be diabetics, according to the consensus criteria [13]. Patients treated for diabetes mellitus, and patients not previously known as diabetics but with a fasting glucose ≥ 125 mg/dL, were considered diabetics, according to international guidelines [13]. During a 2-year period, intra- and postoperative management was similar, except for the blood glucose management: strict glucose control was not implemented until June 2005 and insulin therapy was only initiated after the blood glucose level (BGL) had reached > 150 mg/dL. During surgery, blood glucose measurements were performed after induction, every 30 min during cardiopulmonary bypass. In intensive care, BGLs were controlled every 3 h during the first 12 h after arrival. Afterwards, blood glucose measurements were scheduled every 6 h. From January until May 2005, several different insulin regimens were tested on performance, BGL variability and safety in order to achieve tight glycemic control (80 to 110 mg/dL) with a minimal risk of hypoglycemia. During this period, the Aalst Glycemia Insulin Protocol was conceived, tested, and adjusted to optimize performance [14]. Because of different major adjustments to the insulin protocol during the testing and implementation period, no outcome data were recorded. Only at the end of May 2005 were the performance and safety of the Aalst Glycemia Insulin Protocol were considered satisfactory for general implementation in the cardiac operating theatres and the intensive care unit. From June 2005 onwards, both intra- and postoperative BGL were strictly targeted between 80 to 110 mg/dL using the Aalst Glycemia Insulin Protocol for cardiac surgery [14]. According to the algorithm, blood glucose measurements were scheduled every 30 min intraoperatively and every 60 min in intensive care. This regimen has been described extensively elsewhere [13]. There were no changes in standard operational procedures, both in the operating room and in the ICU. Basic fluid management in the ICU consisted of 1 mL/kg/h of dextrose 5% in both groups. Additional fluid administration with colloids or crystalloids was based on a clinical decision at the discretion of the attending intensivist. The departments of anesthesia, ICU, cardiac surgery, nephrology and perfusion consisted of the same staff members, and no new types of surgery were introduced during the study period. The decision for initiating renal replacement therapy was based on clinical variables at the discretion of the attending nephrologist.

Because of this important change in perioperative care, we were able to study two groups of consecutive patients undergoing cardiac surgery with the use of CPB: in the Control group, operated between August and December 2004, there was no strict blood glucose control, both during surgery as in the ICU. Insulin therapy was only initiated after BGL reached > 150 mg/dL. From June 2005 until June 2006, both intra- and postoperative BGLs were strictly controlled between 80 to 110 mg/dL using the Aalst Glycemia Insulin Protocol in all patients. The conditions and conduct of hypothermic (28°C) CPB remained constant throughout the study period. Myocardial protection was provided by cold antero- and/or retrograde St. Thomas solution in all cases. The Aalst Glycemia Insulin Protocol in all patients was continued in the ICU until enteral feeding was started. Preoperative variables needed for the additive European System for Cardiac Operative Risk Evaluation (EuroSCORE) and Cleveland Clinic Severity Score calculation are shown in Table 2. Data collection fo the Control group consisted of reviewing each patient file separately, and entering all data in a data management file. The data collection of the BGL and outcome in the Insulin group was prospectively designed. Only patients with a complete dataset were included in this analysis.

**Table 2:** Patient characteristics

	Control	Insulin	p Value
Period	August to December 2004	June 2005 to June 2006	
Total (n)	305	745	
Female	103 (33.8)	265 (35.6)	0.61
Body Mass Index, mean ± SD	26.1 ± 4.0	26.0 ± 4.3	0.98
Insulin-treated diabetics, n (%)	17 (5.6)	33 (4.4)	0.43
Non-Insulin-treated diabetics, n (%)	33 (10.8)	89 (11.9)	0.67
Untreated diabetics with fasting glucose ≥ 125 mg/dL, n (%)	22 (7.2)	40 (5.4)	0.25
Fasting glucose (mg/dL)	106 ± 31	107 ± 26	0.11
Unstable angina, n (%)	4 (1.3)	11 (1.5)	1.0
Congestive heart failure, n (%)	48 (15.8)	128 (17.2)	0.65
LVEF 30% to 50%, n (%)	47 (15.6)	99 (13.3)	0.38
LVEF < 30%, n (%)	23 (7.6)	60 (8.1)	0.89
Recent myocardial infarction, n (%)	9 (3)	18 (2.4)	0.67
COPD, n (%)	32 (10.5)	63 (8.4)	0.29
Peripheral arteriopathy, n (%)	5 (1.6)	16 (2.1)	0.80
Neurologic dysfunction, n (%)	19 (6.3)	37 (4.9)	0.45
Serum creatinine > 2.1 mg/dL, n (%)	6 (1.9)	17 (2.3)	1.0
Endocarditis, n (%)	3 (1.0)	8 (1.1)	1.0
Angiotensin-converting Enzyme Inhibitors	105 (34.4)	296 (39.7)	0.12
EuroSCORE, mean ± SD	4 ± 3	4 ± 3	0.76
Cleveland Clinic Severity Score	2 ± 2	3 ± 2	0.19

Data are presented as mean ± SD or number (%) unless otherwise mentioned.

COPD, chronic obstructive pulmonary disease; EuroSCORE, European System for Cardiac Operative Risk Evaluation; LVEF, left ventricular ejection fraction; SD, standard deviation.

The primary endpoint of this retrospective analysis was to evaluate the effect of tight glycemic control on acute renal failure with or without the need for dialysis, in both non-diabetic and diabetic cardiac surgical patients. The degree of renal impairment and/or failure was evaluated using the criteria of the Acute Dialysis Quality Initiative Workgroup [10]. Patients were classified into three severity categories, Risk, Injury and Failure, according to plasma creatinine or estimated glomerular filtration rate and urinary output (Table 1). The estimated glomerular filtration rate was calculated, using the Modification of Diet in Renal Disease equitation [15]. When RIFLE scores based on plasma creatinine, estimated glomerular filtration rate or urinary output were not congruent, the most severe score was recorded. The RIFLE scores 'Loss and End stage renal failure' were not relevant to this analysis because their criteria (renal function loss > 4 weeks) exceeded the study period. RIFLE classification was calculated in the ICU the morning after surgery. Additionally, the maximal RIFLE score during the entire hospital stay was registered. Patients not scoring R, I or F were classified as '0-RIF', patients scoring R, I, or F but without the need for *de novo *postoperative dialysis as 'RIF-D' and patients with renal failure requiring postoperative dialysis as 'RIF+D'. Additionally, to evaluate the effect of tight glycemic control on the expected incidence of acute renal failure requiring dialysis after cardiac surgery, we used the Cleveland Clinic Severity Score [12]. This score predicts the incidence of acute renal failure requiring dialysis across 4 categories of severity, based on an absolute score (0 to 17) using 13 preoperative clinical variables as follows. Scoring 1 point: female gender, congestive heart failure, left ventricular ejection fraction < 35%, chronic obstructive pulmonary disease (COPD), insulin-requiring diabetes, previous cardiac surgery, only valve surgery; scoring 2 points: coronary artery bypass graft (CABG) + valve surgery, other cardiac surgery, emergency surgery, preoperative creatinine 1.2 to < 2.1 mg/dL, preoperative use of intra-aortic balloon pump (IABP); scoring 5 points: preoperative creatinine ≥ 2.1 mg/dL. Patients scoring 0 to 2 points have a predicted incidence for acute postoperative dialysis of 0.4%. A score between 3 to 5 points represents a risk of 1.8%. Patients scoring 6 to 8 points have a risk of 9.5% and patients scoring 9 to 13 points have a predicted incidence of 21.3%.

Secondary endpoints of this retrospective analysis were the effect of tight glycemic control on the incidence of 30-day mortality and in-hospital morbidity in diabetic and non-diabetic cardiac surgical patients. Severe in-hospital morbidity was defined as one or more of (a) cardiac outcome: low cardiac output and/or hypotension treated with an IABP and/or ≥ 2 intravenous inotropes or vasopressors during more than 24 h, malignant arrhythmia (asystole, ventricular tachycardia, or ventricular fibrillation) requiring cardiac resuscitation; (b) respiratory outcome: mechanical ventilation > 48 h, reintubation, tracheotomy; (c) renal outcome: acute renal failure requiring dialysis; (d) infectious outcome: any use of intravenous antibiotics, other than those used for prophylaxis, with of without positive cultures; and (e) other outcome: any surgery or invasive procedure necessary to treat a postoperative adverse event associated with the initial cardiac surgery.

### Statistical analysis

Uni- and multivariate analysis for assessment of the relationships between potential prognostic factors and need for dialysis was performed by using the Fisher exact test, Mann-Whitney U test, analysis of variance (ANOVA), multinomial logistic regression analysis and Student t test when appropriate. Data are expressed as mean ± standard deviation (SD) for continuous variables and numbers and percentages for qualitative variables. All p values were two-tailed. p < 0.05 was considered significant.

## Results

### Preoperative characteristics

**Figure 1:**
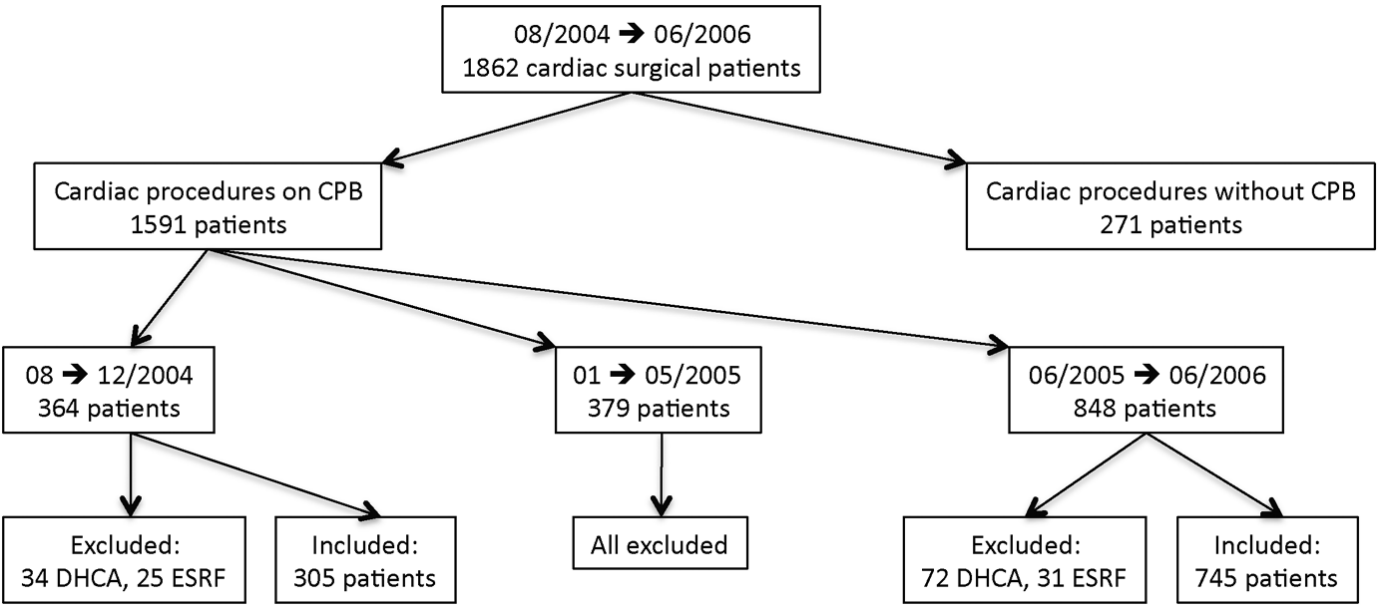
Overview of enrolment process. CPB, cardiopulmonary bypass; DHCA, deep hypothermic circulatory arrest; ESRF, end-stage renal failure.

Of the 1,862 patients scheduled for cardiac surgery between August 2004 and June 2006, a total of 1,050 patients were included in this retrospective analysis, with 305 patients in the Control and 745 patients in the Insulin group (Figure 1). Preoperative demographic data are shown in Table 2. EuroSCORE risk profiles and Cleveland Clinic Severity Scores were similar between groups (not significant).

### Blood glucose control

**Figure 2:**
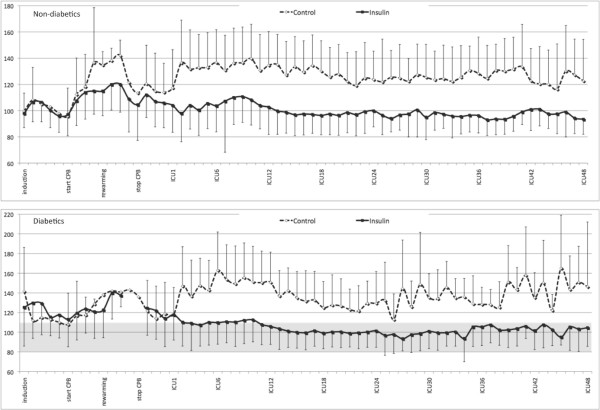
Mean blood glucose levels ± standard deviation (SD) (mg/dL) during surgery and during ICU stay between groups. Induction, startCPB, rewarming, stopCPB, arrival ICU, ICU12, ...24, ...36, ...48 = blood glucose level at induction of anesthesia, on the initiation of cardiopulmonary bypass, at rewarming to normothermia on CPB, at separating from bypass, at admission in the ICU and after 12, 24, 36 and 48 h after arrival in the ICU, respectively. Control, control group; CPB, cardiopulmonary bypass; ICU, Intensive Care Unit; Insulin, group with tight glycemic control.

At induction of anesthesia, mean BGLs of non-diabetics were comparable between groups: 100 ± 14 mg/dL (Control) vs 98 ± 11 mg/dL (Insulin), respectively (p = 0.10). During surgery, from rewarming on CPB onwards, BGLs in the Insulin group were significantly lower than in the Control group at all measured time points until the end of surgery (p < 0.0001; Figure 2). At ICU admission, mean BGL in the Insulin group (104 ± 21 mg/dL) was significantly lower than in the Control group (117 ± 29 mg/dL; p < 0.001). After arrival in the ICU, BGL in the Insulin group remained significantly lower until ICU discharge (p < 0.0001; Figure 2). The preset target of 80 to 110 mg/dL was reached in 71% of all measurements.

In diabetics, mean BGLs at induction of anesthesia were higher in the Control group than in the Insulin group: 142 ± 45 mg/dL vs 125 ± 39 mg/dL, respectively (p = 0.01). Until ICU admission, BGLs were comparable between groups (not significant; Figure 2). Afterwards, mean BGL in the Insulin group remained significantly lower until ICU discharge (p < 0.0001; Figure 2). The preset target of 80 to 110 mg/dL was reached in diabetics in 59.5% of all measurements.

Hypoglycemia (BGL < 50 mg/dL) in the Control group occurred in 9/305 (2.9%) vs 5/745 (0.7%) patients in the Insulin group (p = 0.006).

In the Insulin group, tight glycemic control in the ICU was relatively short with the 10th and 90th percentile at 15.0 and 46.0 h, respectively. As much as 70% of all patients in the Insulin group were in the ICU for 24 h or less and were therefore exposed to tight glycemic control for a limited period of time. For '0-RIF' and 'RIF-D' patients, median duration of tight glycemic control was comparable, 21.0 (9.0 to 48.0) h and 21.0 (10.0 to 48.0) h, respectively (not significant). The median duration of tight glycemic control in the RIF+D patients was 312 (72 to 2,304) h, because of their longer ICU stay.

### Renal function

On the morning after surgery, fewer patients in the Insulin group scored R (11.4 vs 24.4%, p < 0.0001), I (0.9 vs 4.6%, p < 0.003) or F (0.1 vs 1.3%, p < 0.026) than in the Control group.

Maximal RIFLE scores were lower in the Insulin group and there were significantly more '0-RIF' patients in the Insulin group (54.0%) than in the Control group (39.6%) (p < 0.001). Mean creatinine levels in the Control group significantly increased from 0.98 ± 0.41 at admission to 1.23 ± 0.68 mg/dL at hospital discharge in 'RIF-D' patients (p = 0.015). In contrast, in the Insulin group there was no significant change in mean creatinine levels between hospital admission and discharge (1.02 ± 0.36 vs 1.10 ± 0.42 mg/dL, p = 0.12).

In the Control group there were 183 patients who developed renal injury/failure (scoring R, I or F). In 24 (13.1%) of them, renal injury was solely attributable to low urinary output (as defined by the definition of RIFLE), and in 10 (5.5%) solely to an increase in serum creatinine. In the Insulin group there were 342 patients who developed renal injury/failure (scoring R, I or F). In 87 (25.4%) of them, renal injury was solely attributed to low urinary output (as defined by the definition of RIFLE), and in 4 (1.2%) solely to an increase in serum creatinine. Between groups, there were significantly more patients in the Insulin group scoring R, I or F solely based on low urinary output criteria (p = 0.002), but significantly less patients developing renal injury based on an isolated increased serum creatinine (p < 0.001).

**Figure 3:**
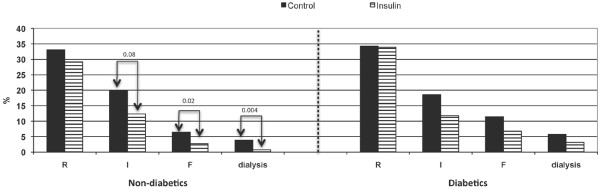
Percentage of patients with R, I or F (according to the RIFLE score) and postoperative dialysis throughout hospital stay, both in non-diabetic and diabetic patients. Control, control group; F, renal failure; I, impairment of renal function; Insulin, group with tight glycemic control; R, risk for renal failure.

In non-diabetics, there were significantly more '0-RIF' patients in the Insulin group (55.7%) then in the Control group (40.4%) (p = 0.002). The incidence in patients maximally scoring R during the entire hospital stay was similar between groups (p = 0.27). In contrast, for non-diabetics maximally scoring I and F, there was a significant difference between groups (p = 0.008 and p = 0.02, respectively) (Figure 3). Moreover, the incidence of acute postoperative dialysis in non-diabetic patients decreased from 3.9% (n = 9) in the Control group to 0.7% (n = 4) in the Insulin group (p = 0.004) (Figure 3).

In diabetics, there was a similar incidence of '0-RIF' patients in both the Insulin and the Control group, 47.5% and 35.7% respectively (p = 0.11). It did not affect postoperative R (p = 1.0), I (p = 0.21) and F (p = 0.27) scoring (Figure 3), or the incidence of acute postoperative dialysis (p = 0.45).

**Figure 4:**
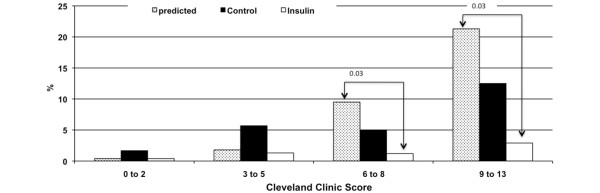
Comparison of the predicted vs the observed incidence of acute renal failure with the need for dialysis in non-diabetics between groups. 0 to 2, 3 to 5, ... represent the different risk classes for acute renal failure with dialysis, as defined by the Cleveland Clinic Severity Score: 0 to 2 representing a predicted incidence of ARF with dialysis of 0.4%, 3 to 5 representing a predicted incidence of ARF with dialysis of 1.8%, 6 to 8 representing a predicted incidence of ARF with dialysis of 9.5%, 9 to 13 representing a predicted incidence of ARF with dialysis of 21.3%. Control, control group; Insulin, group with tight glycemic control; predicted, the predicted risk for postoperative dialysis based on the Cleveland Clinic Severity Score.

The distribution into different risk classes of the predictive Cleveland Clinic Severity Score was comparable between groups (not significant). The observed overall incidence of acute postoperative dialysis was adequately predicted by the Cleveland Clinic Severity Score in the Control group (p = 0.6), but was 60% lower in the Insulin group than predicted (1.2 vs 3%, p = 0.03). In non-diabetics, the observed vs predicted incidence of acute postoperative dialysis in the severe (6 to 8 points) to high (9 to 13 points) risk classes was significantly lower in the Insulin group (severe risk: 1.2% vs 9.5%, p = 0.03; high risk: 2.9% vs 21.3%, p = 0.03) (Figure 4). The difference between the predicted vs observed incidence of postoperative dialysis in the Control group was not significant (1.8 vs 5.7%, p = 0.21). In diabetics, there was no significant difference between the predicted vs observed incidence of acute postoperative dialysis throughout all risk classes (not significant).

Results from multinomial regression analysis on preoperative angiotensin-converting enzyme inhibitors and perioperative aprotinin administration and packed cell transfusion are represented in Table 3. The preoperative use of angiotensin-converting enzyme inhibitors was not associated with a preoperative increased serum creatinine > 1.5 mg/dL in neither groups (p = 1.0 (Control); p = 0.75 (Insulin)).

**Table 3:** Multinomial logistic analysis

	RIF		Survival	
		
	OR (CI)	p Value	OR (CI)	p Value
Control group:				
Non-diabetics				
Aprotinin	0.9 (0.4 to 2.2)	0.79	2.3 (0.5 to 11.2)	0.30
ACE inihibitors	1.0 (0.6 to 1.8)	0.94	0.7 (0.2 to 2.8)	0.60
Transfusion	0.9 (0.5 to 1.7)	0.79	0.8 (0.1 to 5.9)	0.69
Diabetics				
Aprotinin	0.9 (0.3 to 2.8)	0.94	0.5 (0.2 to 1.4)	0.32
ACE inihibitors	1.4 (0.5 to 3.6)	0.52	12.6 (1.4 to 109.2)	0.02
Transfusion	1.6 (0.5 to 5.9)	0.4	0.7 (0.07 to 7.3)	0.77
Insulin group:				
Non-diabetics				
Aprotinin	0.7 (0.4 to 1.1)	0.14	2.0 (0.5 to 9.7)	0.39
ACE inihibitors	0.4 (0.3 to 0.5)	0.001	0.7 (0.2 to 3.1)	0.70
Transfusion	0.7 (0.5 to 1.1)	0.15	1.0 (0.6 to 2.9)	0.58
Diabetics				
Aprotinin	1.0 (0.3 to 2.8)	0.97	1.7 (0.3 to 5.8)	0.26
ACE inihibitors	1.2 (0.7 to 2.3)	0.65	3.4 (1.2 to 12.3)	0.001
Transfusion	1.5 (0.8 to 3.0)	0.25	1.0 (0.8 to 1.1)	0.92

ACE, angiotensin-converting enzyme; CI, confidence interval; OR, odds ratio; RIF, renal failure according to RIFLE (Risk of renal failure, Injury to kidney function, Failure of kidney function, Loss of kidney function and End-stage renal failure) score criteria.

### Postoperative morbidity and 30-day mortality

The distribution of procedures is shown in Table 4. In the Insulin group, patients suffered significantly less cardiac (p < 0.0001), renal (p = 0.003) and infectious (p = 0.003) morbidities than in the Control group. Length of ICU stay was comparable between groups (p = 0.78), as well as the use of intravenous diuretics in the ICU (p = 1.0).

**Table 4:** Perioperative data

Group	Subgroup	Control	Insulin	p Value
Study interval		August to December 2004	June 2005 to June 2006	
Procedure, n (%):	CABG	97 (31.8)	218 (29.3)	0.41
	Valve	132 (43.3)	291 (39.0)	0.21
	Combined	76 (24.9)	236 (31.7)	0.03
	Redo	43 (14.2)	104 (13.9)	1.0
	Emergency	33 (10.9)	61 (8.2)	0.19
ICU length of stay (days), median (min-max)		2 (1 to 73)	2 (1 to 106)	0.61
Aprotinin usage, n (%)		29 (9.5)	103 (13.8)	0.06
Administration of intravenous diuretics into the ICU, n(%)		50 (16.4)	121 (16.2)	1.0
Patients with packed cell transfusion, n (%)		240 (78.7)	524 (70.3)	0.006
Packed cells per patient, median (min-max)		2 (0 to 18)	2 (0 to 19)	0.66
Blood glucose level (mg/dL):	Induction	109 ± 39	104 ± 24	0.20
	End of surgery	115 ± 25	108 ± 22	< 0.001
	Admission to ICU	118 ± 29	107 ± 22	< 0.001
	Mean in the ICU	133 ± 29	103 ± 15	< 0.0001
Serum creatinine levels (g/dL):				
Non-diabetics	Preoperative	1.0 ± 0.7	1.0 ± 0.5	0.99
	Max in the ICU	1.3 ± 1.0	1.1 ± 0.6	0.09
	Max post ICU	1.0 ± 1.0	0.9 ± 0.8	0.33
Diabetics	Preoperative	1.0 ± 0.3	1.2 ± 1.1	0.11
	Max in the ICU	1.3 ± 1.2	1.4 ± 1.0	0.31
	Max post ICU	1.2 ± 1.7	1.3 ± 1.4	0.57
Morbidity, n (%):				
Non-diabetics	Cardiac	56 (24.0)	62 (10.6)	< 0.0001
	Renal	9 (3.9)	4 (0.7)	< 0.01
	Pulmonary	7 (3.0)	38 (6.5)	0.06
	Infectious	23 (9.9)	25 (4.3)	< 0.01
	Other	9 (3.9)	24 (4.1)	1.0
Diabetics	Cardiac	22 (31.4)	46 (28.4)	0.64
	Renal	4 (5.7)	5 (3.1)	0.45
	Pulmonary	6 (8.6)	15 (9.3)	1.0
	Infectious	5 (7.1)	7 (4.3)	0.35
	Other	2 (2.9)	8 (4.9)	0.72
30-day Mortality, n (%):				
Non-diabetics		7 (3.0)	5 (0.9)	< 0.05
Diabetics		4 (5.6)	4 (2.5)	0.25
Cause of death, n (%):				
Cardiac		4 (36.4)	2 (22.2)	0.64
Respiratory		0 (0.0)	1 (11.1)	0.45
Sepsis		1 (9.1)	1 (11.1)	1.0
Multi organ failure		5 (45.4)	4 (44.4)	1.0
Other		1 (9.1)	1 (11.1)	1.0

Data are presented as mean ± SD or number (%) unless otherwise mentioned.

CABG, coronary artery bypass graft; ICU, Intensive Care Unit; SD, standard deviation.

**Figure 5:**
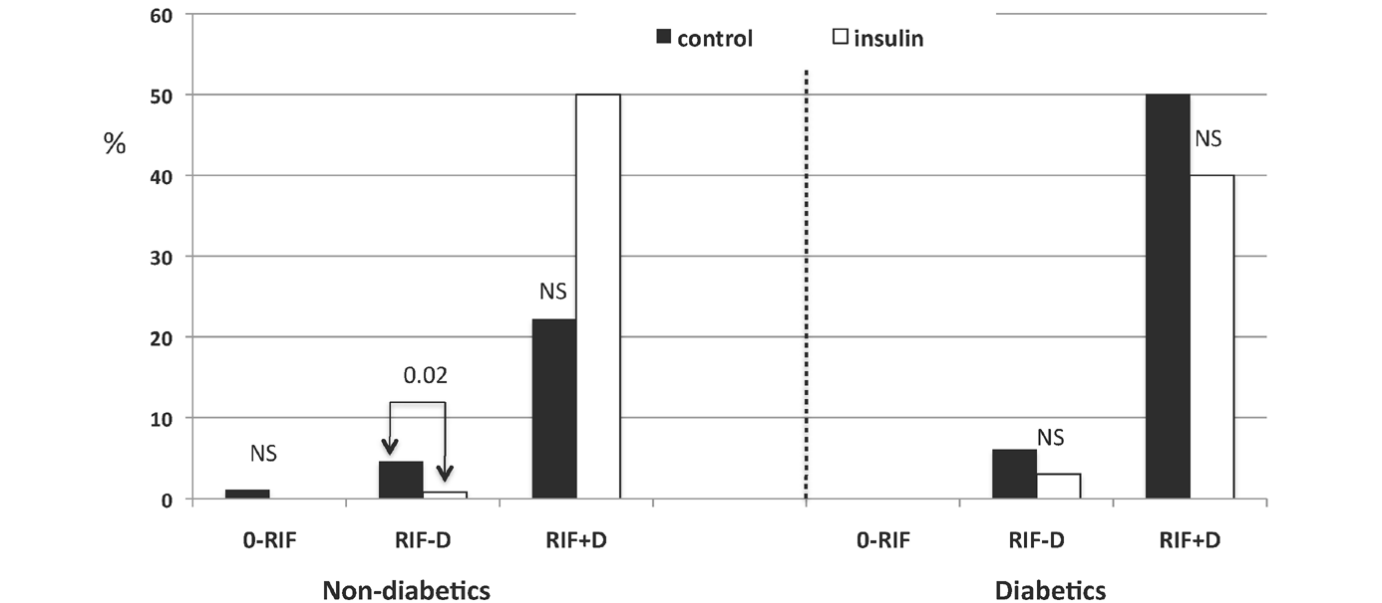
Comparison of 30-day mortality between groups. 0-RIF, patients without R, I or F score; RIF-D = patients scoring R(isk), I(mpairment) or F(ailure) (accoding to the RIFLE score) but without the need for haemodialysis; RIF+D, patients requiring hemodialysis. Control, control group; Insulin, group with tight glycemic control.

The overall incidence of 30-day mortality was significantly lower in the Insulin group than in the Control group (1.2 vs 3.6%, respectively) (p = 0.02). In non-diabetics, 30-day mortality decreased from 3.0% in the Control group to 0.9% in the Insulin group (p < 0.05), representing a relative reduction of 70.0%. In diabetics, the incidence was 5.6% in the Control and 2.5% in the Insulin group (p = 0.25), representing a relative reduction of 56.1% (Table 3). Figure 5 compares the incidence of 30-day mortality between both groups in both non-diabetic and diabetic '0-RIF', 'RIF-D' and 'RIF+D' patients. In non-diabetics, the 30-day mortality in 'RIF-D' patients was significantly lower in the Insulin group than in the Control group (0.8 vs 4.6%) (p = 0.02).

## Discussion

The risk of renal injury and/or failure after cardiac surgery varies between 5% to 30% and 1% to 5% of cardiac surgical patients develop renal failure requiring dialysis [1]. This widely varying incidence of renal failure is related to the heterogeneity in study design and end points [16]. Recently, the RIFLE score has been proposed as consensus criteria of the Acute Dialysis Quality Initiative Workgroup [15] and has been validated in cardiac surgery [11]. Because RIFLE provides a uniform clinical definition for renal failure, we used this scoring system as a sensitive tool to evaluate the effect of tight perioperative BGL control. In the present study, tight perioperative glycemic control is associated with a significant reduction in severe RIFLE scoring.

Although our study design does not allow to conclude that intraoperative tight glucose control as such has nephroprotective effects, it should be noted that by introducing intraoperative BGL control, intraoperative hyperglycemia, an independent risk factor for mortality [6], is avoided. Furthermore, BGL at ICU admission, a surrogate of subsequent glucose control [17], is lower. By already imposing tight glycemic control with insulin in the operating theatre, the target of BGL control in the ICU is reached more quickly [14] as compared to other studies that focused only on postoperative BGL control [4,18]. Some previous work has suggested that intraoperative BGL control does not contribute to postoperative outcome [7]. If so, then our results would be remarkable in the sense that, for patients scoring R, I or F, a relatively short period of postoperative BGL control would have such an effect on renal failure (90% of patients were treated with the Aalst Glycemia Insulin Protocol in the ICU for less than 46.0 h). Other groups have argued that the beneficial effects of insulin are related to its anti-inflammatory and antioxidant properties rather than tight glycemic control. It has been shown that 2 h of insulin administration (2 IU/h) has similar anti-inflammatory effects as 100 mg hydrocortisone intravenously [19]. In patients with acute myocardial infarction, low-dose insulin has anti-inflammatory, antioxidant and pro-fibrinolytic effects, independently of a decrease in blood glucose levels [20]. In cardiac surgical patients, C-reactive protein concentrations decrease during high-dose insulin infusion but increase within hours after insulin withdrawal [21]. However, Van den Berghe *et al. *have reported that the improvement in outcome with low-dose insulin infusion depends more on the reduction in plasma glucose levels than on the dose of insulin administered in critically ill patients [22].

Implementing tight glycemic control is associated with an increased risk of hypoglycemia. A recent meta-analysis in critically ill patients has demonstrated that intensive insulin therapy is associated with a sixfold increase in the relative risk of hypoglycemia [23] and as much as 5.1 to 17.0% of patients develop glucose levels < 40 mg/dL [4,24]. Such incidence and levels of hypoglycemia may mask potential benefits of perioperative glucose control and were considered crucial to stop ongoing trials early [24,25]. However, in a previous study with the Aalst Glycemia Insulin Protocol, a dynamic algorithm that adapts insulin dosage to intrinsic insulin sensitivity and changes in BGL over time [14], hypoglycemia (BGL < 50 mg/dL) occurred only in 0.7% of patients, with 40 mg/dL as lowest value. The incidence of hypoglycemia in this study is comparable (0.7% of patients with BGL < 50 mg/dL).

It should be noted that in a recent meta-analysis including 29 studies (8,432 patients), tight glucose control was not associated with a significant reduction in hospital mortality or in new need for hemodialysis [26]. However, the authors report a markedly increased risk for hypoglycemia and in 21% of the studies mean glucose target was not reached within 5 mg/dL. Whether these 2 factors affect a potential benefit of tight glycemic control is a matter of speculation. Because the combination of poorly performing algorithms and hypoglycemia is the Achilles' heel of tight glycemic control, prospective randomized trials using an algorithm that combines both adequate tight glucose control with a minimal risk for hypoglycemia may provide answers to these questions.

To the best of our knowledge, this is the first study that evaluates the effect of perioperative glycemic control in cardiac surgery, comparing the observed incidence of dialysis with that predicted by the Cleveland Clinic Severity Score [12]. Although tight glycemic control did not reduce the incidence of dialysis in patients at low risk, it successfully reduced the need for dialysis in the severe (-87.5%) and high (-86.5%) risk non-diabetic patients, as compared to the predicted incidence. In patients at risk for acute kidney injury (that is, patients scoring R, I or F but without the need for dialysis), mean creatinine level between hospital admission and discharge increased by 25% in the Control group but not in the Insulin group. Even a slight increase in serum creatinine (0.5 mg/dL) after cardiac surgery predisposes to increased mortality [27]. Whether this is clinically relevant in a cardiac surgical setting is still unknown. Also, the reason for the decreased risk on renal failure in non-diabetics using angiotensin-converting enzyme inhibitors is unknown to us. Similarly, the apparently improved survival of diabetics using angiotensin-converting enzyme inhibitors is remarkable. Whether this is clinically relevant is unknown to us at this time, and our database does not allow us to draw long-term conclusions due to the limited number of diabetics in this analysis.

Mortality rates in excess of 50% have been reported in cardiac surgical patients requiring dialysis [1,4]. Avoiding the need for renal replacement therapy is probably a key factor in reducing mortality. The observed 60% reduction in postoperative dialysis in our Insulin group may have contributed to decreased mortality rates. However, in patients that need hemodialysis, tight glycemic control did not reduce mortality. The fact that relatively short-term tight glycemic control during and after cardiac surgery has such an impact on renal function and mortality is new, and to a certain extent in contrast to the findings of Zerr and Furnary who showed beneficial effects after 48 h [28,29]. This concept was confirmed by Van den Berghe, showing that tight glycemic control is beneficial for patients staying 3 days or more in the ICU [30]. However, we have previously demonstrated that intraoperative tight glycemic control results in postoperative mean BGL between 80 to 110 mg/dL within 1 h after ICU admission, and in a low BGL variability [14]. The latter has been reported to be an independent predictor of ICU and hospital mortality [31]. Consequently, even patients staying in the ICU for a short period of time would benefit from tight glycemic control. But in another single-centre trial, tight glycemic control during cardiac surgery had no effect on the incidence of renal failure [7]. However, in that study, mean BGL did not reach the preset target of 80 to 100 mg/dL during surgery as well as in theICU. It is possible that this observation, together with the fact that 15.0% of patients in the control group did receive insulin, masks the potential benefit of tight intraoperative glycemic control in their investigation.

Finally, in our analysis, we acknowledge that statistical power is not sufficient to draw conclusions on the effect of tight glycemic on in-hospital outcome in diabetics. They suffer from a chronic state of insulin resistance, with inhibition of the insulin-signaling cascade by free fatty acids [32] or by inflammatory cytokines, such as TNFα [33]. Short-term insulin treatment may not be able to overcome these metabolic derangements and fail to protect renal function.

Another limitation is that, although the data collection for the Aalst Glycemia Insulin Protocol in all patients was prospectively designed, the comparison between the Control and Insulin groups was not randomized. Therefore this analysis remains retrospective in nature. However, the Cleveland Clinic Severity Score is a prospective score. The comparison of the observed vs predicted incidence of acute renal failure requiring dialysis is not affected by the retrospective nature of this study. The findings of our retrospective analysis may be controversial with respect to the time frame of tight glycemic control. However, one could speculate that the beneficial effects of tight glycemic control in patients that stay in the ICU for a relatively short period of time may be attributed to in part by strict intraoperative BGL control. Therefore, a prospective study is needed to validate this concept.

## Conclusion

Tight intra- and postoperative glucose control with intravenous insulin is associated with a significant reduction in postoperative renal impairment and injury according to the RIFLE criteria in non-diabetic cardiac surgical patients. Despite of a relatively short ICU-stay, the need for acute postoperative dialysis, as well as 30-day mortality were significantly lower in non-diabetics benefiting from tight glucose control.

## Key messages

• In non-diabetic cardiac surgical patients, tight blood glucose control is associated with a decreased incidence of renal impairment and postoperative dialysis according to RIFLE criteria, as well as 30-day mortality.

• Improved renal outcome associated with tight glycaemic control is present even after a relatively short ICU stay.

• In diabetic cardiac surgical patients, tight glycaemic control is not associated with improved renal outcome or 30-day mortality.

• The observed overall incidence of acute postoperative dialysis in patients with perioperative tight glycaemic control is lower than predicted by the Cleveland Clinic Severity Score.

## Abbreviations

BGL: blood glucose level; CPB: cardio pulmonary bypass; ETCO_2_: end tidal carbon dioxide; ICU: intensive care unit; MAC: minimal alveolar concentration; OR: operating room.

## Competing interests

The authors declare that they have no competing interests.

## Authors' contributions

All authors actively participated in this study, read the manuscript and attest to the validity and legitimacy of the data and its interpretation.

## References

[B1] Chertow G, Levy E, Hammermeister K, Grover F, Daley J (1998). Independent association between acute renal failure and mortality following cardiac surgery. Am J Med.

[B2] Lok C, Austin P, Wang H, Ju T (2004). Impact of renal insufficiency on short-term and long-term outcomes after cardiac surgery. Am Heart J.

[B3] Ouattara A, Lecomte P, Le Manach Y, Landi M, Jacqueminet S, Platonov I, Bonnet N, Riou B, Coriat P (2005). Poor intraoperative blood glucose control is associated with a worsened hospital outcome after cardiac surgery in diabetic patients. Anesthesiology.

[B4] Berghe G Van den, Wouters P, Weekers F, Verwaest C, Bruyninckx F, Schetz M, Vlasselaers D, Ferdinande P, Lauwers P, Bouillon R (2001). Intensive insulin therapy in critically ill patients. N Engl J Med.

[B5] Furnary AP, Gao G, Grunkemeier GL, Wu Y, Zerr KJ, Bookin SO, Floten HS, Starr A (2003). Continuous infusion reduces mortality in patients with diabetes undergoing coronary artery bypass grafting. J Thorac Cardiovasc Surg.

[B6] Doenst T, Wijeysundera D, Karkouti K, Zechner C, Maganti M, Rao V, Borger M (2005). Hyperglycemia during cardiopulmonary bypass is an independent risk factor for mortality in patients undergoing cardiac surgery. J Thorac Cardiovasc Surg.

[B7] Ghandi G, Nuttall G, Abel M, Mullany C, Schaff H, O'Brien P, Johnson M, Williams A, Cutshall S, Mundy L, Rizza R, Mc Mahon M (2007). Intensive intraoperative insulin therapy versus conventional glucose management during cardiac surgery. Ann Intern Med.

[B8] Han H, Lee Y, Park S, Lee J, Taub M (2005). High glucose-induced oxidative stress inhibits Na^+^/glucose cotransporter activity in renal proximal tubule cells. Am J Physiol Renal Physiol.

[B9] Baines A, Ho P (2002). Glucose stimulates O_2 _consumption, NOS and Na^+^/H exchange in diabetic rat proximal tubules. Am J Physiol Renal Physiol.

[B10] Bellomo R, Ronco C, Kellum J, Metha R, Pavelsky P, the ADQI workgroup (2004). Acute Renal failure – definition, outcome measures, animal models, fluid therapy and information technology needs: the Second International Consensus Conference on the Acute Dialysis Quality Initiative (ADQI) Group. Crit Care.

[B11] Kuitunen A, Vento A, Suojaranta-Ylinen R, Petilla V (2006). Acute renal failure after cardiac surgery: evaluation of the RIFLE classification. Ann Thorac Surg.

[B12] Thakar C, Arrigain S, Worley S, Yared J-P, Paganini E (2005). A clinical score to predict acute renal failure after cardiac surgery. J Am Soc Nephrol.

[B13] Genuth S, Alberti KG, Bennett P, Buse J, Defronzo R, Kahn R, Kitzmiller J, Knowler WC, Lebovitz H, Lernmark A, Nathan D, Palmer J, Rizza R, Saudek C, Shaw J, Steffes M, Stern M, Tuomilehto J, Zimmet P, Expert Committee on the Diagnosis and Classification of Diabetes Mellitus (2003). Follow-up report on the diagnosis of diabetes mellitus. Diabetes Care.

[B14] Lecomte P, Foubert L, Nobels F, Coddens J, Nollet G, Casselman F, Van Crombrugge P, Vandenbroucke G, Cammu G (2008). Dynamic tight glycemic control during and after cardiac surgery is effective, feasible and safe. Anesth Analg.

[B15] Levey A, Bosch J, Lewis J, Greene T, Rogers N, Roth D (1999). A more accurate method to estimate glomerular filtration rate from serum creatinine: a new prediction equation. Modification of Diet in Renal Disease Study Group. Ann Intern Med.

[B16] Zacharias M, Gilmore I, Herbison G, Sivalingam P, Walker R (2005). Interventions for protecting renal function in the perioperative period. Cochrane Database Syst Rev.

[B17] Egi M, Bellomo R, Stachowski E, French G, Hart G, Stow P (2006). Blood glucose on day of intensive care unit admission as a surrogate of subsequent glucose control in intensive care. J Crit Care.

[B18] Goldbergh P, Sakharova O, Barrett P, Falko L, Roussel M, Bak L, Blake-Holmes D, Marieb N, Inzucchi S (2004). Improving glycemic control in cardiothoracic intensive care unit: clinical experience in two hospital setting. J Cardiothorac Vasc Anesth.

[B19] Dandona P, Thusu K, Hafeez R, Abdel-Rahman E, Chaudhuri A (1998). Effect of hydrocortisone on oxygen free radical generation by mononuclear cells. Metabolism.

[B20] Chaudhuri A, Janicke D, Wilson M, Tripathy D, Garg R, Bandyopadhyay A, Calieri J, Hoffmeyer D, Syed T, Ghanim H, Aljada A, Dandona P (2004). Anti-inflammatory and pro-fibrinolytic effect of insulin in acute ST-elevation myocardial infarction. Circulation.

[B21] Visser L, Zuurbier J, Hoek F, Opmeer B, de Jonge E, de Mol B, van Wezel H (2005). Glucose, insulin and potassium applied as perioperative hyperinsulinaemic normoglycaemic clamp: effects on inflammatory response during coronary artery surgery. Br J Anaesth.

[B22] Vanhorebeek I, De Vos R, Mesotten D, Wouter P, De Wolf P, Berghe G Van den (2005). Protection of hepatocyte mitochondrial ultrastructure and function by strict blood glucose control with insulin in critically ill patients. Lancet.

[B23] Thomas G, Rojas M, Epstein S, Balk E, Liangos O, Jaber B (2007). Insulin therapy and acute kidney injury in critically ill patients – a systematic review. Nephrol Dial Transplant.

[B24] Brunkhorst F, Engel C, Bloos F, Meier-Hellmann A, Ragaller M, Weiler N, oerer O, Gruendling M, Oppert M, Grond S, Olthoff D, Jaschinski U, John S, Rossaint R, Welt T, Schaefer M, Kern P, Kuhnt E, Kiehntopf M, Hartog C, Natason C, Loefler M, Reinhart K, German Competence Network Sepsis (SepNet) (2008). Intensive Insulin Therapy and Pentastarch Resuscitation in severe sepsis. N Engl J Med.

[B25] Chaney M, Nikolov M, Blakeman B, Bakhos M (1999). Attempting to maintain normoglycemia during cardiopulmonary bypass with insulin may initiate postoperative hypoglycemia. Anesth Analg.

[B26] Wiener R, Wiener D, Larson R (2008). Benefits and risks of tight glucose control in critically ill adults. JAMA.

[B27] Lassnigg A, Schmidlin D, Mouhieddine M, Bachmann L, Druml W, Bauer P, Hiesmayr M (2004). Minimal changes of serum creatinine predit prognosis in patients after cardiothoracic surgery: a prospective cohort study. J Am Soc Nephrol.

[B28] Zerr K, Furnary A, Grinkemeier G, Bookin S, Kanhere V, Starr A (1997). Glucose control lowers the risk of wound infection in diabetics after open heart operations. Ann Thorac Surg.

[B29] Furnary A, Zerr K, Grunkemeier G, Starr A (1999). Continuous intravenous insulin infusion reduces the incidence of deep sternal wound infection in diabetic patients after cardiac surgical procedures. Ann Thorac Surg.

[B30] Berghe G Van den, Wilmer A, Hermans G, Meerseman W, Wouters P, Milants I, Van Wijngaerdeen E, Bobbaers H, Bouillon R (2006). Intensive insulin therapy in the medical ICU. N Engl J Med.

[B31] Egi M, Bellomo R, Stachowski E, French C, Hart G (2006). Variability of blood glucose concentration and short-term mortality in critically ill patients. Anesthesiology.

[B32] Yu C, Chen Y, Cline G, Zhang D, Zong H, Wang Y, Bergeron R, Kim J, Cushman S, Cooney G, Atcheson B, White M, Kraegen E, Shulman G (2002). Mechanism by which fatty acids inhibit insulin activation of insulin receptor substrate-1 (IRS-1)-associated phosphatidylinositol 3-kinase activity in muscle. J Biol Chem.

[B33] Hatanaka E, Monteagudo P, Marrocos M, Campa A (2006). Neutrophils and monocytes as potentially important sources of proinflammatory cytokines in diabetes. Clin Exp Immunol.

